# Utilization of convolutional neural networks to analyze microscopic images for high-throughput screening of mesenchymal stem cells

**DOI:** 10.1515/biol-2022-0859

**Published:** 2024-07-10

**Authors:** MuYun Liu, XiangXi Du, JunYuan Hu, Xiao Liang, HaiJun Wang

**Affiliations:** National Engineering Research Center of Foundational Technologies for CGT Industry, Shenzhen, Guangdong, China; Shenzhen Cellauto Automation Co., Ltd., Shenzhen, Guangdong, China; Shenzhen Beike Biotechnology Co., Ltd., Shenzhen, Guangdong, China

**Keywords:** microscopic images, separable convolutional neural network, human bone marrow mesenchymal stem cells, classification accuracy

## Abstract

This work investigated the high-throughput classification performance of microscopic images of mesenchymal stem cells (MSCs) using a hyperspectral imaging-based separable convolutional neural network (CNN) (H-SCNN) model. Human bone marrow mesenchymal stem cells (hBMSCs) were cultured, and microscopic images were acquired using a fully automated microscope. Flow cytometry (FCT) was employed for functional classification. Subsequently, the H-SCNN model was established. The hyperspectral microscopic (HSM) images were created, and the spatial-spectral combined distance (SSCD) was employed to derive the spatial-spectral neighbors (SSNs) for each pixel in the training set to determine the optimal parameters. Then, a separable CNN (SCNN) was adopted instead of the classic convolutional layer. Additionally, cultured cells were seeded into 96-well plates, and high-functioning hBMSCs were screened using both manual visual inspection (MV group) and the H-SCNN model (H-SCNN group), with each group consisting of 96 samples. FCT served as the benchmark to compare the area under the curve (AUC), *F*1 score, accuracy (Acc), sensitivity (Sen), specificity (Spe), positive predictive value (PPV), and negative predictive value (NPV) between the manual and model groups. The best classification Acc was 0.862 when using window size of 9 and 12 SSNs. The classification Acc of the SCNN model, ResNet model, and VGGNet model gradually increased with the increase in sample size, reaching 89.56 ± 3.09, 80.61 ± 2.83, and 80.06 ± 3.01%, respectively at the sample size of 100. The corresponding training time for the SCNN model was significantly shorter at 21.32 ± 1.09 min compared to ResNet (36.09 ± 3.11 min) and VGGNet models (34.73 ± 3.72 min) (*P* < 0.05). Furthermore, the classification AUC, *F*1 score, Acc, Sen, Spe, PPV, and NPV were all higher in the H-SCNN group, with significantly less time required (*P* < 0.05). Microscopic images based on the H-SCNN model proved to be effective for the classification assessment of hBMSCs, demonstrating excellent performance in classification Acc and efficiency, enabling its potential to be a powerful tool in future MSCs research.

## Graphical abstract

**Figure:**
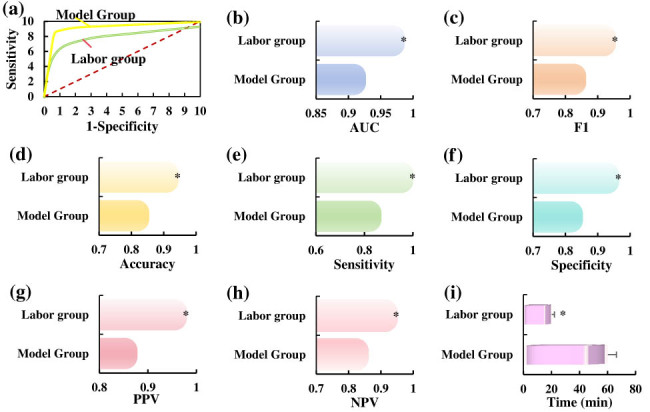

## 1 Introduction

In modern biomedical research, mesenchymal stem cells (MSCs) have garnered significant attention [1]. They possess essential characteristics such as self-renewal, immunomodulation, and multi-lineage differentiation, and are widely distributed in various sources, including bone marrow, adipose tissue, and placenta [2,3]. Clinical studies have indicated the potential of MSCs in tissue engineering, regenerative medicine, and immunotherapy [4,5,6]. It is worth noting that MSCs from different sources exhibit distinct characteristics; for instance, adipose-derived MSCs primarily focus on self-renewal and differentiation [7], umbilical cord MSCs are mainly associated with proliferation and immunomodulation [8], and bone marrow MSCs primarily emphasize multi-lineage differentiation [9]. Quality control is of paramount importance in working with MSCs, as poor quality can lead to less effective treatments or adverse reactions [10,11].

Traditional methods for screening MSCs involve laboratory techniques such as flow cytometry (FCT) or immunohistochemistry. However, these methods are time-consuming, labor-intensive, and require specialized expertise, rendering them unsuitable for high-throughput screening [12,13]. Recently, microscopy analysis has emerged as a highly promising approach for evaluating MSCs’ characteristics [14]. Microscopic images can provide information about cell morphology, distribution, structure, quantity, and functionality, simplifying the differentiation of various MSC types [15]. Nevertheless, manually counting and classifying MSCs through visual inspection often suffer from low efficiency and inadequate classification accuracy (Acc), posing challenges in delivering timely results for clinical applications.

The rapid evolution of artificial intelligence technologies has led to the widespread use of deep learning algorithms for image classification and processing. Within this landscape, the rapid development of convolutional neural networks (CNN) has opened up possibilities for the classification of cells in microscopic images [16]. Experts have already applied CNN for classifying microscopic images, such as using CNN models for microalgae microscopic image classification or using Faster R-CNN and deep CNN for the classification of multi-stage mitotic cell classification and detection [17,18]. CNN has become an essential tool in the field of biomedical image analysis. CNN not only enhances feature extraction and classification capabilities but also possesses the ability to automatically learn [19,20]. As a result, CNN can automatically learn the morphological features of images, facilitating the efficient processing of large-scale image data, thus avoiding the inefficiencies associated with manual feature extraction. This positions CNN as an ideal tool for processing microscopic images [21,22]. Kim et al. [23] confirmed that deep learning models represent a convenient high-throughput method for evaluating the classification efficacy of MSCs and can be used as an effective quality control method in future clinical bio-manufacturing processes. However, traditional CNN methods in the classification of microscopic images only capture information related to cell colors, lacking insight into their underlying biochemical characteristics. As a solution, experts have proposed combining the “all-in-one” characteristic of microscopic images with CNN for cell microscopy image classification. Research has demonstrated that a CNN model incorporating the “spectrum-all-in-one” feature of hyperspectral imaging can not only comprehensively capture information in microscopic cell images but also rapidly and accurately analyze a large number of cell images. Furthermore, it possesses automatic learning capabilities, reducing manual intervention and simplifying the processing, thus positively impacting the advancement of clinical biomanufacturing and cell research [24].

In summary, this work represented the inaugural application of the hyperspectral imaging-based separable CNN (H-SCNN) model combined with hyperspectral imaging technology for the analysis of microscopic images of MSCs, assessing the model’s classification performance on MSCs. The aim of this work is to develop an effective screening method that can automatically learn and extract morphological features from images, thereby mitigating the inefficiencies of manual feature extraction, by harnessing the biological characteristics of MSCs and the computational capabilities of H-SCNN. This empowers clinicians to rapidly and accurately identify MSCs with specific characteristics, promoting further progress in stem cell research and providing robust support for the clinical applications, drug discovery, and fundamental research related to MSCs.

## 2 Research methods

### 2.1 Cell culture

In this work, Human bone marrow mesenchymal stem cell (hBMSCs) were sourced from Guangzhou GeniBio Biotechnology Co., Ltd, and were cultivated *in vitro* for subsequent investigations. The *in vitro* cultivation of hBMSCs typically necessitates specialized culture media and conditions to maintain their growth and functionality [25]. The specific cultivation method was as follows:

First, the culture medium was prepared, which involved using Dulbecco’s Modified Eagle’s Medium/Ham’s F-12 (DF12), obtained from Guangdong EnviroBio Technology Co., Ltd, as the basal culture medium. In addition, 10–20% fetal bovine serum from Thermo Fisher Scientific, China was incorporated into the medium, followed by the addition of 1% l-glutamine (Jiangsu Pules Biological Technology Co., Ltd) and antibiotics, typically 100 IU/mL of penicillin and 100 μg/mL of streptomycin (Beijing Soleibao Technology Co., Ltd). Subsequently, cell cultivation was initiated: hBMSCs were placed into culture dishes (Thermo Fisher Scientific, China), covered with sterile coverslips from the same source, and incubated in an environment maintained at 37°C with 5% CO_2_ gas for 2 weeks. During this period, the culture medium was refreshed every 2–3 days. Growth of hBMSCs was periodically observed to ensure they exhibited their typical fibroblast-like morphology. When the cell density reached a certain level, typically at 80–90% confluence, cell passaging was performed to separate and redistribute hBMSCs into new culture dishes to increase the cell population.

### 2.2 Acquisition and processing of microscopic images

Under the controlled conditions of 37°C with 5% CO_2_, the microscopic imaging of hBMSCs cells was observed using an automated microscope provided by Meigu Molecular Instruments (Shanghai) Co., Ltd. Microscopic images were captured using phase objectives (40× and 100×). A total of 3,200 8-bit grayscale images were collected and were subjected to adjustment based on the hue (*H*), saturation (*S*), and value (*V*) of the image to minimize their impact on the experiments. Images with an average *V* value exceeding 240 were excluded because excessively high brightness could cause cell boundaries to merge with the background, making differentiation challenging. The average *V* value of the remaining images was adjusted to approximately 130. Subsequently, the image size was resized to 220 × 300 pixels using interpolation techniques available in the Python OpenCV Toolbox. Ultimately, 1,400 microscopic images were obtained and utilized for subsequent research.

### 2.3 FCT

Following the acquisition of microscopic images, the research collected corresponding cells for FCT to assess the expression levels of the surface antigens CD73 and CD90. First, hBMSCs were carefully gathered and rinsed with phosphate-buffered saline from Sigma-Aldrich to eliminate culture media and impurities. The cell count was determined using the Countstar, fully automated cell counter from Shanghai Ruiyu Biotech Co., Ltd, and a cell suspension was prepared, maintaining a concentration ranging from 1–5 × 10^6^ cells/mL. Next the required number of cells was taken and placed in Nunc 1.5 mL centrifuge tubes from Thermo Fisher Scientific, China. Subsequently, the cell suspension was combined with fluorescein isothiocyanate (FITC)-labeled CD73 antibody and phycoerythrin (PE)-labeled CD90 antibody from Shanghai Ruiyu Biotech Co., Ltd, both at a concentration of 10 μg/mL. The cells and antibodies were mixed and incubated at 4°C for 30 min. To eliminate unbound antibodies, samples were washed with fluorescence-activated cell sorting (FACS) buffer from Thermo Fisher Scientific, China and then subjected to a 5-min centrifugation at 1,500 rpm for discharging the supernatant. FACS buffer was added to the cell pellet, and the cells were suspended. Flow cytometric analysis of the cell samples was performed using the CytoFLEX S flow cytometer from Beckman Coulter International Trading (Shanghai) Co., Ltd. The instrument was configured to excite and detect FITC and PE fluorescence signals. By detecting the fluorescence signal of each cell, the FCT could determine whether CD73 and CD90 were expressed on the cell surface. The data obtained were subsequently analyzed using DIVA software to gauge the expression levels of CD73 and CD90, providing valuable insights into the cellular properties of hBMSCs. High functionality was defined as CD73 and CD90 positive expression levels exceeding 95%, while lower levels were categorized as indicating reduced functionality.

### 2.4 Model establishment

#### 2.4.1 Construction of hyperspectral microscopic (HSM) images

**Figure 1:**
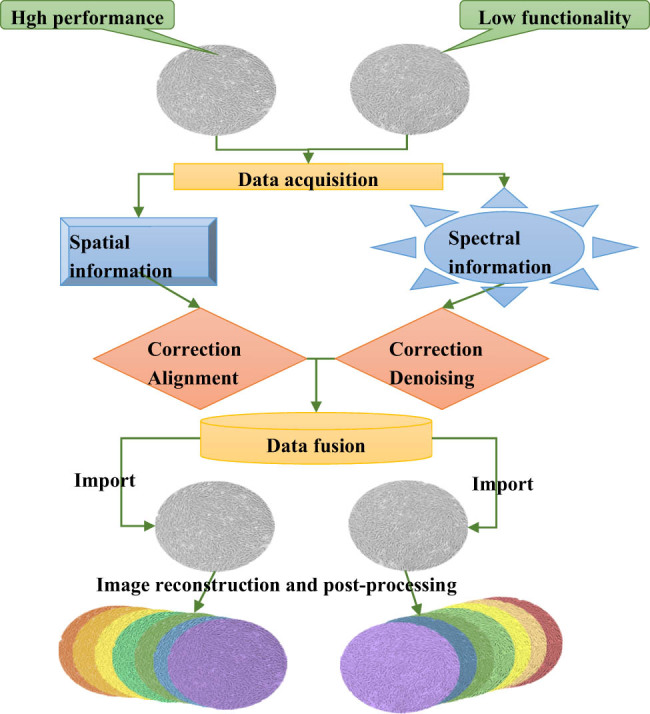
Construction of HSM images.

The construction of HSM imaging involved leveraging spatial-spectral feature (SSF) information from hyperspectral images to enhance the classification efficacy of microscopic images. Constructing SSF-based microscopic images is an image processing technique that combines spectral and spatial data, typically employed in fields such as materials science and biology. This technology aids in the identification and analysis of the composition, distribution, and properties of different materials or substances. The general steps for constructing SSF-based microscopic images are illustrated in Figure 1. First, the data were acquired. In this work, the hBMSCs properties were assessed using FCT to distinguish between high-performance and low-performance hBMSCs within the microscopic images. Spectral and spatial information for both types of cells were then collected to facilitate the classification. Subsequently, data were preprocessed, including correcting and denoising spectral information and aligning and correcting spatial information to correspond with spectral information. After that, spectral data were merged with spatial information. Finally, microscopic images were constructed. The fused data were adopted to construct microscopic images, with the aid of interpolation techniques available in the Python OpenCV Toolbox for image reconstruction. Furthermore, additional steps such as denoising, enhancement, and contrast adjustment were also carried out.

Image denoising: (a) the median filtering denoising algorithm was selected to mitigate noise in the image; (b) denoising parameters were fine-tuned to balance denoising effectiveness and the preservation of image details; (c) image denoising tools were employed to apply the selected denoising method for noise reduction.

Image enhancement: (a) image contrast was enhanced initially to highlight target features and reduce background interference; (b) brightness and saturation were adjusted to improve the visual quality of the image; (c) histogram equalization or other enhancement techniques were utilized to optimize the image’s histogram distribution.

Contrast adjustment: (a) image editing tools or dedicated image processing software (such as Adobe Photoshop) were employed to adjust the image’s contrast to ensure that target features were more clearly visible; (b) linear or nonlinear contrast adjustment methods can be employed based on specific requirements; (c) whether the adjusted image meets the analysis or visualization needs was evaluated, making iterative adjustments as per the specific application.

**Figure 2:**
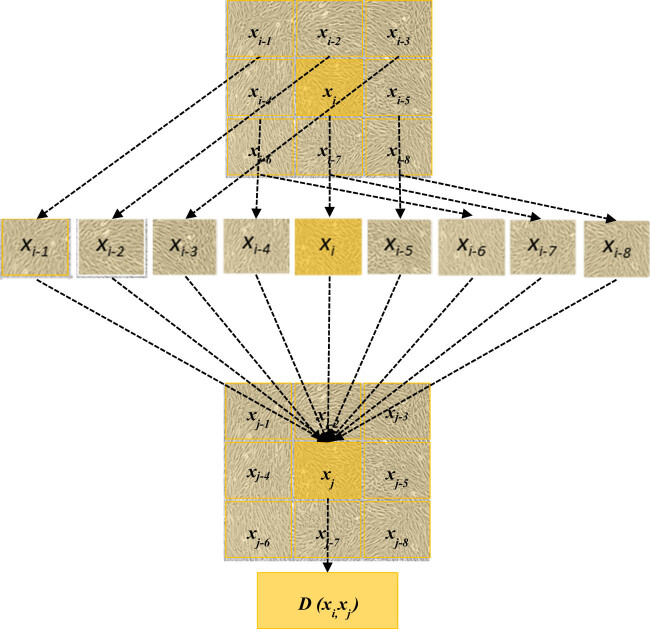
Calculation process of SSCD.

HSM images exhibited a noticeable spatial correlation among pixel distributions, with pixels in close spatial proximity tending to share the same characteristics [26,27]. In this work, spatial-spectral neighbors (SSNs) were selected based on the similarity of joint spatial-spectral information in the neighborhood. This approach can help increase the training samples, as depicted in Figure 2. It was assumed that the dataset of HSM images was represented as \[X\text{}=\text{}{[}{x}_{1},\text{}{x}_{2},\text{}\mathrm{..}.,\text{}{x}_{i}]]\] , where *X* constituted the data matrix and *x*_*i*_ referred to the spectral vector of the *i*th pixel. Taking the example of pixels *x*_*i*_ and *x*_*j*_ in HSM images of MSCs, their neighboring spatial regions were represented as *S*(*x*_*i*_) and *S*(*x*_*j*_), respectively, and the spatial-spectral combined distance SSCD could be expressed as follows:

(1) \[D({x}_{i},\text{}{x}_{j})=D(S({x}_{i})+S({x}_{j})).]\]

In the above equation, \[D(S({x}_{i})+S({x}_{j}))]\] referred to the distance from the pixels *x*_*i*_ to *x*_*j*_ in the neighboring spatial regions *S*(*x*_*i*_) and *S*(*x*_*j*_), respectively.

#### 2.4.2 SCNN

SCNN, commonly abbreviated as depthwise separable convolution or depthwise separable ConvNet, is a CNN architecture frequently used for image processing and computer vision tasks [28]. The SCNN structure maintains model performance while reducing the number of parameters, thereby lowering computational costs and memory consumption [29].

SCNN is composed of two principal components: depthwise convolution (DC) and pointwise convolution (PC) [30].

DC: In traditional convolutional operations, each input channel undergoes convolution with a convolutional kernel, yielding a single output channel. However, DC is the first step in separable convolution, enabling each input channel to be convolved independently with its respective convolutional kernel, generating output channels equal in number to the input channels, without mixing information between channels. In this work, it was considered that the input feature map possessed C channels and the size of the convolutional kernel is \[K\times K\hspace{.03em}]\] . In this case, DC can be calculated as follows:

For each channel c:

Input of the feature map: *I*_c_, with the size of \[H\times W\hspace{.03em}]\] (*H* denotes the height and *W* denotes the weight);

Convolution kernel: *K*_c_, \[K\times K\hspace{.03em}]\] ;

Output of the feature map: *O*_c_, \[(H-K+1)\times ]\] \[(W-K+1)]\] ;

DC can be calculated with following equation:

(2) \[{O}_{\text{c}}{[}i,j]=\text{sum}({I}_{\text{c}}{[}i:i+K,\text{}j:j+K]\ast {K}_{\text{c}})\text{for}\hspace{.5em}i=0\hspace{.25em}\text{to}\hspace{.25em}H-K,\text{}j=0\hspace{.25em}\text{to}\hspace{.25em}W-K.]\]

PC: It is the second step in separable convolution and involves traditional 1 × 1 convolution. It was employed to condense the quantity of output channels from the DC to the desired number. PC convolved the output of DC using a 1 × 1 convolutional kernel to generate the final output. The specific calculation method for PC is as follows:

It was assumed that there were *E* output channels for DC:

Input of the feature map: the output *O*_c_ of DC, with the size of \[(H-K+1)\times (W-K+1)]\] ;

Convolution kernel: there were *E* convolution kernels with a size of 1 × 1, representing as *K*1, *K*2,…, *KD*;

Output of the feature map: the final output feature map, \[(H-K+1)\times (W-K+1)]\]

Calculation expression of PC is given in equation (3).

(3) \[E{[}i,j]=\text{sum}({O}_{\text{c}}i{[}i,j]\ast {K}_{\text{i}})\hspace{.25em}\text{for}\hspace{.25em}i=0\hspace{.25em}\text{to}\hspace{.25em}H-K,\text{}j=0\hspace{.25em}\text{to}\hspace{.25em}W-K\hspace{.03em}.]\]

In the above expression, \[E{[}i,j]]\] refers to each channel of the output feature map and \[{O}_{\text{c}}i]\] denotes the *i*th output channel of the DC.

**Figure 3:**
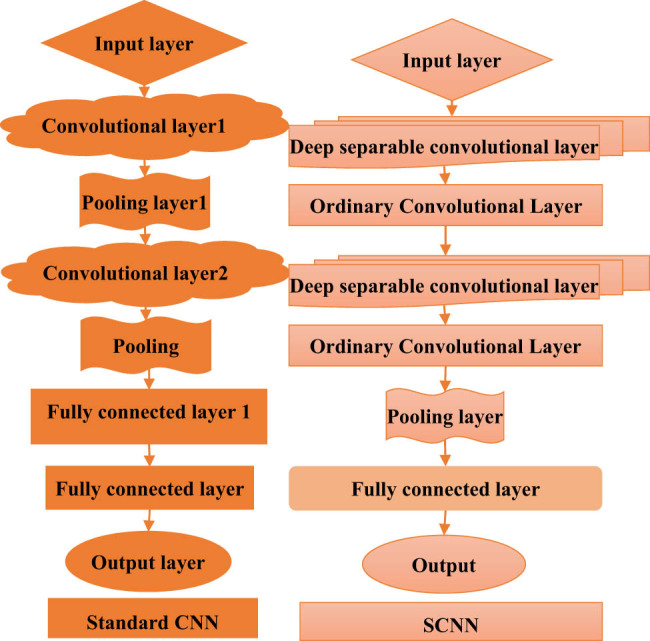
Comparison between standard CNN and SCNN.

Difference between CNN and SCNN is illustrated in Figure 3.

#### 2.4.3 Evaluation methods

This work aimed to assess the effectiveness of the methods employed for classifying HSM images. To achieve this, an initial training dataset comprising of 1,400 microscopic images was applied to obtain the optimal parameter samples for the H-SCNN model. Subsequently, sample sets of sizes 20, 30, 40, 50, 60, 70, 80, 90, and 100 were each selected for analysis to evaluate the classification Acc of SCNN and other CNN models (using FCT detection results as the reference standard). These additional CNN models primarily included well-known ResNet and VGGNet models. Concurrently, the training times required for various CNN models were compared.

### 2.5 Cell grouping

In Section 2.1, this work involved seeding cultured cells into a 96-well culture plate. Subsequently, an automated microscope was utilized to observe the hBMSCs present in each well. Following this, high-functioning hBMSCs were screened through two distinct approaches: manual visual inspection (MV group) and the H-SCNN model. These two screening methods were designated as the MV group and the H-SCNN group, respectively, each consisting of 96 samples. The evaluation of both screening methods was performed, with FCT detection results serving as the reference standard.

### 2.6 Observation parameters

Furthermore, the analysis effectiveness of classification methods for hBMSCs in different groups was compared using distinct metrics, including area under the curve (AUC), *F*1 score, Acc, sensitivity (Sen), specificity (Spe), positive predictive value (PPV), and negative predictive value (NPV). Additionally, the time differences between distinct classification methods were observed to identify an efficient and effective screening method with strong classification efficacy.

(4) \[\text{Acc}=\frac{\text{TP}+\text{TN}}{\text{TP}},]\]

(5) \[\text{Sen}=\frac{\text{TP}}{\text{TP}+\text{FN}},]\]

(6) \[\text{Spe}=\frac{\text{TN}}{\text{TN}+\text{FP}},]\]

(7) \[\text{PPV}=\frac{\text{TP}}{\text{TP}+\text{FP}},]\]

(8) \[\text{NPV}=\frac{\text{TN}}{\text{TN}+\text{FN}}.]\]

where TP represents the number of samples that are actually positive and correctly predicted as positive by the classifier; TN refers to the number of samples that are actually negative and correctly predicted as negative by the classifier; FP signifies the number of samples that are actually negative but incorrectly predicted as positive by the classifier; and FN indicates the number of samples that are actually positive but incorrectly predicted as negative by the classifier.

### 2.7 Methods for statistical analysis

Data were processed using SPSS 26.0. Continuous data were displayed as mean value ± standard deviation and were compared using the *t*-test. Categorical data were presented as frequencies or percentages (%) and were compared using the χ^2^ test. *P* < 0.05 was considered statistically significant.

## 3 Results

### 3.1 Construction of HSM images and classification efficacy

**Figure 4:**
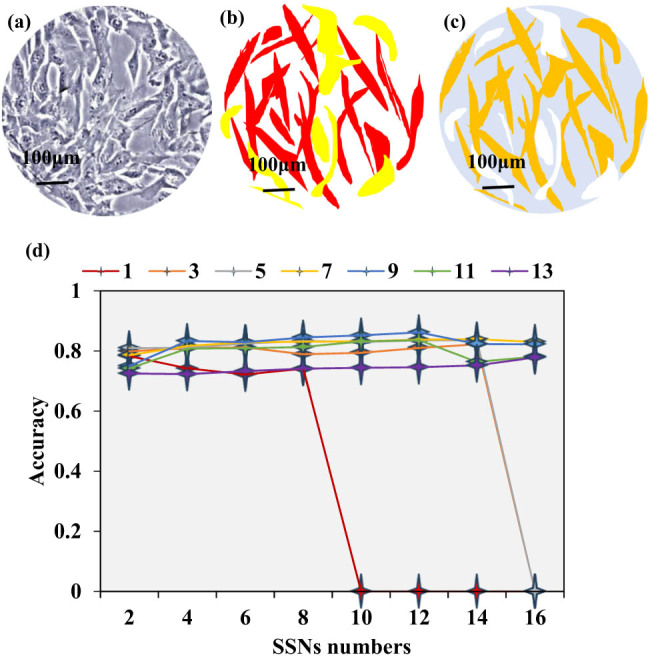
Distribution of hBMSCs and classification Acc: (a) original image; (b) FCT results; (c) hyperspectral hBMSCs microscopic truth map; and (d) results with WS of WS 1, 3, 5, 7, 9, 11, and 13, respectively.

Based on the FCT results, the different functional levels of hBMSCs were labeled in the corresponding microscopic images. White represented the background, red indicated high functionality, and yellow represented low functionality. Simultaneously, the distribution of hBMSCs in the corresponding hyperspectral microscopy ground truth images was observed, where blue signified the background, orange indicated high functionality, and white represented low functionality. Through comparative observations, the distribution of hBMSCs in both scenarios was found to be quite consistent, as displayed in Figure 4a–c. To obtain the optimal algorithm parameters, this work further compared the classification Acc under different Window size (WS) and SSNs numbers. WS was selected from 1, 3, 5, 7, 9, 11, and 13, while SSN numbers were chosen sequentially from 2, 4, 6, 8, 10, 12, 14, and 16. The classification Acc is shown in Figure 4d. It was found that when WS was set to 5–11, Acc was higher, and when the number of SSNs was 8–12, Acc was higher. When WS was set to 9 and the number of SSNs was 12, the classification Acc was 0.862, reaching the highest, indicating that it was the best result. In addition, these parameters were also the basis for subsequent research.

### 3.2 Classification performance of various CNN models

**Figure 5:**
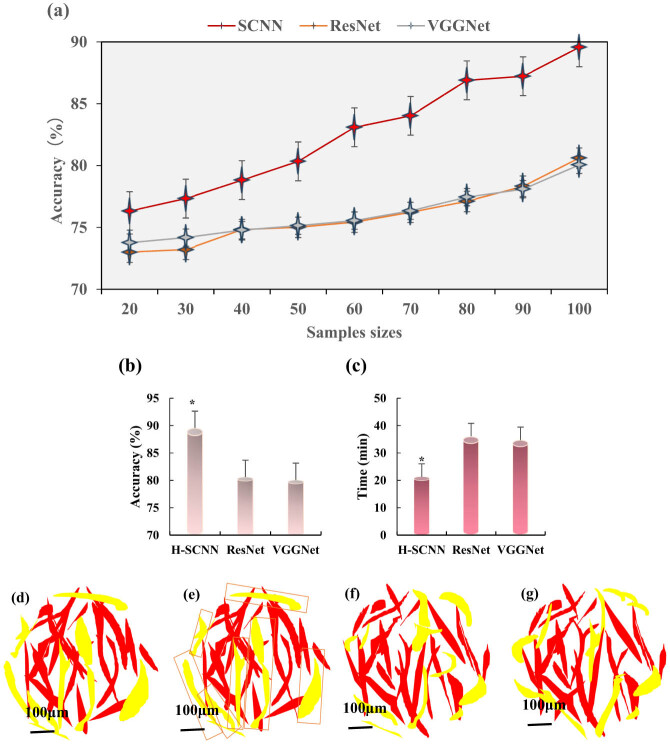
Comparison of classification performance of SCNN, ResNet, and VGGNet models. (a) Acc of SCNN, ResNet, and VGGNet models, respectively; (b) Acc when the sample size was 100; (c) training time; (d) ground truth image; (e) SCNN model; (f) ResNet model; (g) VGGNet model; “*” indicated a statistical significance (*P* < 0.05) compared to ResNet and VGGNet models.

Sample sizes of 20, 30, 40, 50, 60, 70, 80, 90, and 100 microscopic images were employed for training to compare the classification Acc of the hyperspectral hBMSCs microscopic images among the SCNN, ResNet, and VGGNet models. Additionally, the classification efficiencies of the three models were evaluated. As the sample size increased, the classification Acc of all three models gradually increased (Figure 5a). When the sample size reached 100, each model obtained the highest classification Acc. The classification Acc of SCNN, ResNet, and VGGNet models were 89.56 ± 3.09, 80.61 ± 2.83, and 80.06 ± 3.01%, respectively. The classification efficiency of the SCNN model was much higher than that of the ResNet and VGGNet models (*P* < 0.05) (Figure 5b). Figure 5c shows that the training time of SCNN, ResNet, and VGGNet models was 21.32 ± 1.09, 36.09 ± 3.11, and 34.73 ± 3.72 min, respectively. Compared to ResNet and VGGNet models, SCNN models had a shorter training time (*P* < 0.05). Figures 5d–g represent the classification power diagram, indicating that the SCNN model exhibited significantly superior classification performance and was more similar to ground real images.

### 3.3 Comparison on screening efficacy in the H-SCNN and MV groups

**Figure 6:**
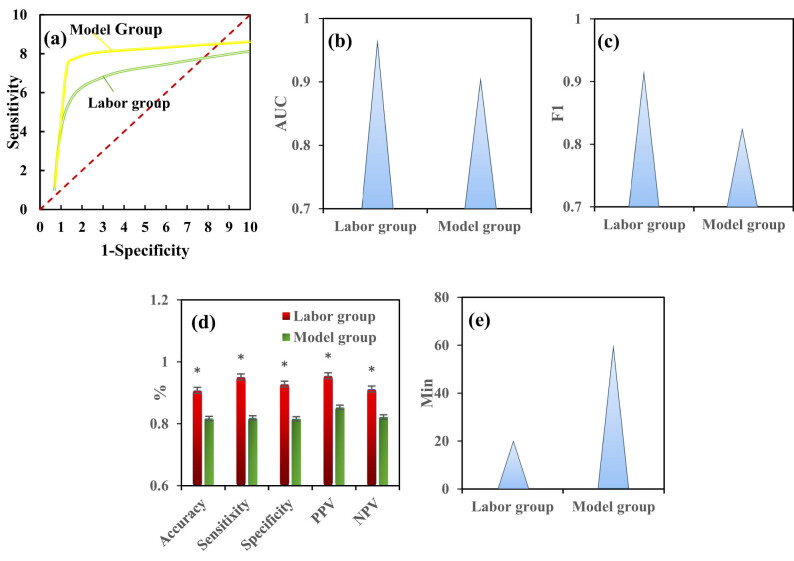
Classification efficacy for hBMSCs in MV and H-SCNN groups. (a) ROC curve; (b) AUC; (c) *F*1 score; (d) Acc, Sen, Spe, PPV, and NPV; (e) time. Note: * suggested a substantial difference with *P* < 0.05 in contrast to the MV group.

In this work, several metrics, including AUC, *F*1 score, Acc, Sen, Spe, PPV, and NPV, were selected to analyze the classification efficacy of hBMSCs in both the MV group and H-SCNN group. Figure 6a displays the ROC curve. According to the ROC analysis, the AUC, *F*1 score, Acc, Sen, Spe, PPV, and NPV for hBMSCs classification in the MV group were 0.908, 0.826, 0.817, 0.819, 0.816, 0.853, and 0.822, respectively. For the H-SCNN group, the corresponding values were 0.968, 0.918, 0.908, 0.951, 0.928, 0.955, and 0.912, respectively. Comparatively, the classification AUC, *F*1 score, Acc, Sen, Spe, PPV, and NPV for the H-SCNN group were all higher than those for the MV group, exhibiting obvious differences (*P* < 0.05), as explicated in Figure 6d. Furthermore, it was observed that the MV group required 60.28 ± 4.16 min to classify 96 microscopic images, whereas the H-SCNN group completed the task in only 20.11 ± 2.17 min, which was obviously faster (*P* < 0.05), as depicted in Figure 6e.

## 4 Discussion

In this work, FCT was utilized to assess the levels of CD73 and CD90 in hBMSCs. CD73, also known as 5′-nucleotidase, is a surface molecule typically expressed in BMSCs. Its primary role involves the conversion of adenosine monophosphate into adenosine on the cell surface, thereby regulating immune responses and cell signal transduction [31]. CD90, also known as Thy-1 or THY1, is a common marker for BMSCs and serves as a surface antigen. It is often utilized for identifying and isolating BMSC populations [32]. Based on the evaluation of functional levels of hBMSCs from the test results, the corresponding hBMSCs in the microscopic images were classified as high-functioning or low-functioning. Once the data were collected, the HSM images were constructed, and the optimal algorithm parameters were determined by comparing the classification Acc under various WS and SSN values. The results suggested that the best parameter combination was WS = 9 and SSN = 12, which achieved a classification Acc of 0.862, making it the best parameter combination for this study. This finding underscores the importance of parameter selection for accurate classification and provides a strong benchmark for subsequent research.

Based on the results, this work further compared the performance of the SCNN, ResNet, and VGGNet models in classifying high-spectral hBMSCs microscopic images. These three models are all commonly utilized in deep learning, are variants of CNN, and are constructed with components like convolutional layers, pooling layers, and fully connected layers for tasks such as image classification and feature extraction. However, they differ in network depth, the number of parameters, and their suitability for various tasks [33,34,35]. VGGNet is relatively shallow, featuring either 16 or 19 convolutional layers and a large number of parameters. ResNet, on the other hand, is very deep, typically having 50, 101, or even more convolutional layers, but fewer parameters compared to VGGNet. In contrast, SCNN is a specialized CNN designed for semantic segmentation, typically consisting of convolutional and deconvolutional layers for pixel-level labeling. It usually falls between VGGNet and ResNet in terms of the number of parameters. While the first two are often used for image classification tasks, SCNN excels in assigning each pixel in an image to a specific category and is typically used for image segmentation tasks. All three have found applications in cell classification studies [36], but this work represented the first comparison of their classification performance. SCNN is a neural network architecture specifically designed for image segmentation tasks, often used to segment different cell structures or nuclei in cell images [37]. In cell classification, SCNN can be used to locate and segment cell nuclei and other cellular components, providing valuable data for subsequent classification tasks to achieve more accurate cell classification and identification [38]. Given that hyperspectral images often contain a substantial number of parameters, efficient training is a key challenge. SCNN excels in handling high-dimensional, large-scale hyperspectral data. Its architectural design effectively reduces the number of model parameters, enhances feature extraction, mitigates overfitting, and improves computational efficiency [39,40]. This work revealed that the SCNN model achieved the highest classification Acc, significantly outperforming the performance of the ResNet and VGGNet models. Furthermore, the training time required for the SCNN model was notably lower in contrast to the other two models. These findings indicate the advantages of the H-SCNN model in terms of classification Acc and efficiency.

In conclusion, the H-SCNN model and MV group methods were adopted to classify high and low functional hBMSCs. The results demonstrated the superiority of the H-SCNN group over the MV group in terms of classification AUC, *F*1 score, Acc, Sen, Spe, PPV, and NPV. Additionally, the H-SCNN group required significantly less time compared to the MV group. This further emphasizes the clear advantages of the H-SCNN model in both classification performance and efficiency. Manual cell classification often relies on the subjective judgment and expertise of trained biologists or medical professionals. Acc can be influenced by subjective factors, leading to potential errors. Moreover, manual classification is labor-intensive and can significantly impact processing speed when dealing with large datasets [41]. In contrast, machine learning algorithms can be trained on extensive and well-labeled datasets, enabling rapid and highly accurate classification [42]. Therefore, the H-SCNN model offers distinct advantages over manual methods. Lyu et al. [43] and Honrado et al. [44] have also proposed through their research that machine learning methods offer greater speed, efficiency, and consistency in cell classification. Lien et al. [45] proposed a multi-layer tensor model, which is an improved CNN that can classify cells derived from induced pluripotent stem cells and evaluate their differentiation efficiency. This model demonstrated the ability to classify MSCs, retinal ganglion cells, and retinal pigment epithelial cells with an Acc of 97.8%. Additionally, it demonstrated the potential to identify candidate cells with ideal characteristics while excluding cells with immature/abnormal phenotypes. Wang et al. [46] proposed an analysis method based on cell physical characteristics and a deep learning method for identifying cell types. By analyzing the processed image using an optimized CNN, two sets of cells (HL-7702 and SMMC-7721, SGC-7901 and GES-1) can be identified. The results showed that using deep learning technology to recognize the physical characteristics of cells can be a universal and effective automatic analysis method for cell information. It is evident that machine learning-based cell classification is typically faster, more consistent, and adaptable, making it particularly well-suited for large-scale cell classification tasks.

## 5 Conclusion

In conclusion, the results and discussions presented above clearly demonstrated the effectiveness of utilizing HSM images and machine learning models for the classification of hBMSCs. In particular, the H-SCNN model exhibited outstanding performance in terms of classification Acc and efficiency, positioning it as a powerful tool for future MSCs research. This work yielded strong support and methods for further exploration of the biological characteristics and clinical applications of MSCs. However, it is essential to acknowledge that the success of these machine learning models hinges on the availability of a substantial amount of labeled data and the fine-tuning of algorithms. Manual cell classification, though time-consuming and subjective, remained useful in certain cases, particularly in scenarios where there was insufficient training data available for machine learning or when complex cell classification situations require the expertise of human professionals. Therefore, in the field of machine learning-based cell classification, researchers should direct their efforts toward refining deep learning algorithms to reduce the reliance on a large amount of labeled data. Techniques such as transfer learning, weakly supervised learning, and self-supervised learning can help enhance algorithm generalization, thus reducing the need for labeled data. This avenue of research holds the potential to further advance the field of automated cell classification while maintaining the flexibility and expertise of human judgment when needed.
