# Corrigendum to “Utilization of convolutional neural networks to analyze microscopic images for high-throughput screening of mesenchymal stem cells”

**DOI:** 10.1515/biol-2025-0001

**Published:** 2025-04-09

**Authors:** MuYun Liu, XiangXi Du, JunYuan Hu, Xiao Liang, HaiJun Wang

**Affiliations:** National Engineering Research Center of Foundational Technologies for CGT Industry, Shenzhen, Guangdong, China; Shenzhen Cellauto Automation Co., Ltd., Shenzhen, Guangdong, China; Shenzhen Beike Biotechnology Co., Ltd., Shenzhen, Guangdong, China

In the published manuscript, “Utilization of convolutional neural networks to analyze microscopic images for high-throughput screening of mesenchymal stem cells. Open Life Sciences. 2024;19(1):20220859. https://doi.org/10.1515/biol-2022-0859,” the authors found an error in graphical abstract and Figure 6b–e. Specifically, the labor group and model group have been inversely labeled.

The authors acknowledge the error and state that it was unintentional, unrelated to any academic misconduct, and does not affect the conclusions of the publication. The authors would like to sincerely apologize to the editor, the journal staff, and the readers for the oversight and any inconvenience it may have caused.

**Figure 1:**
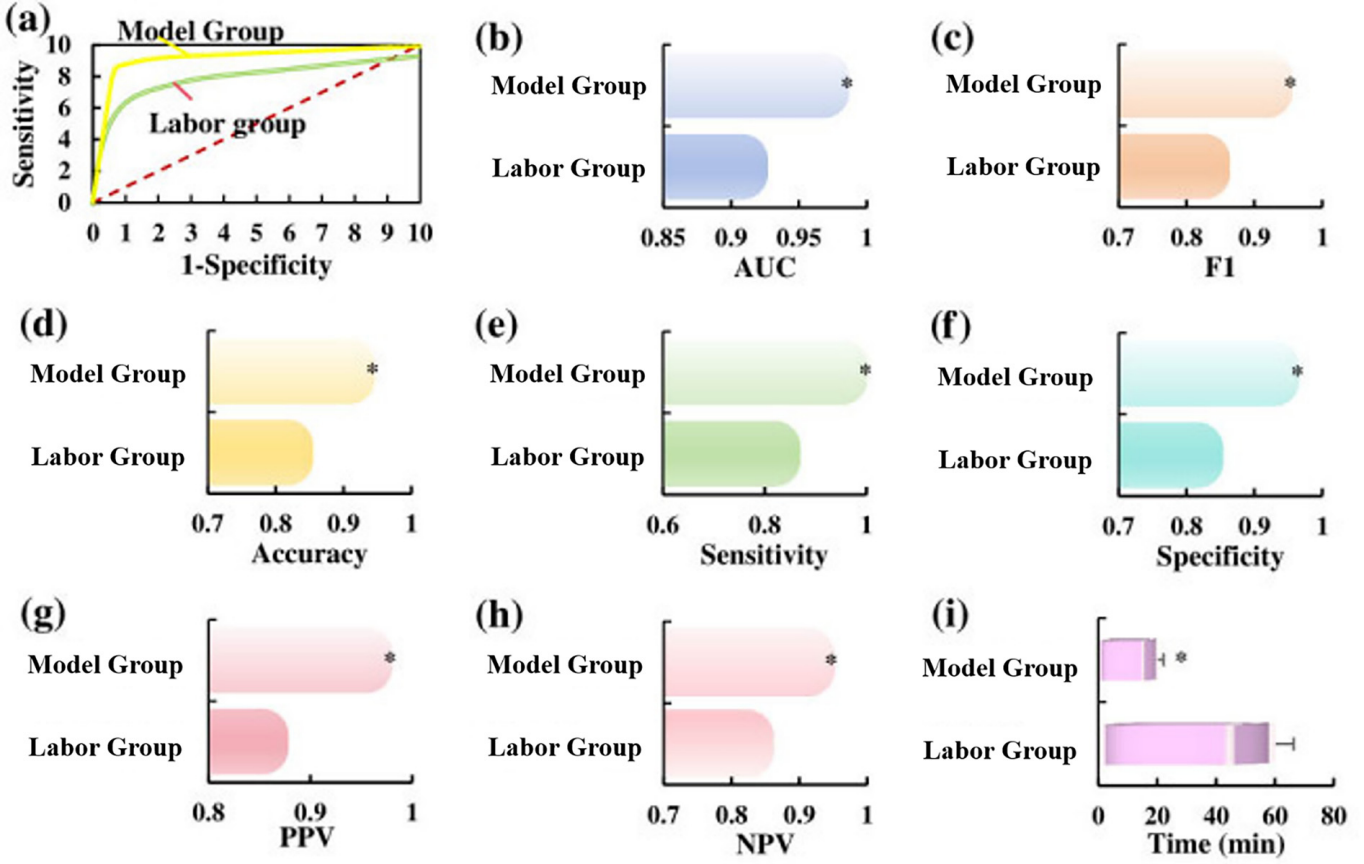
Corrected version of the graphical abstract.

**Figure 2:**
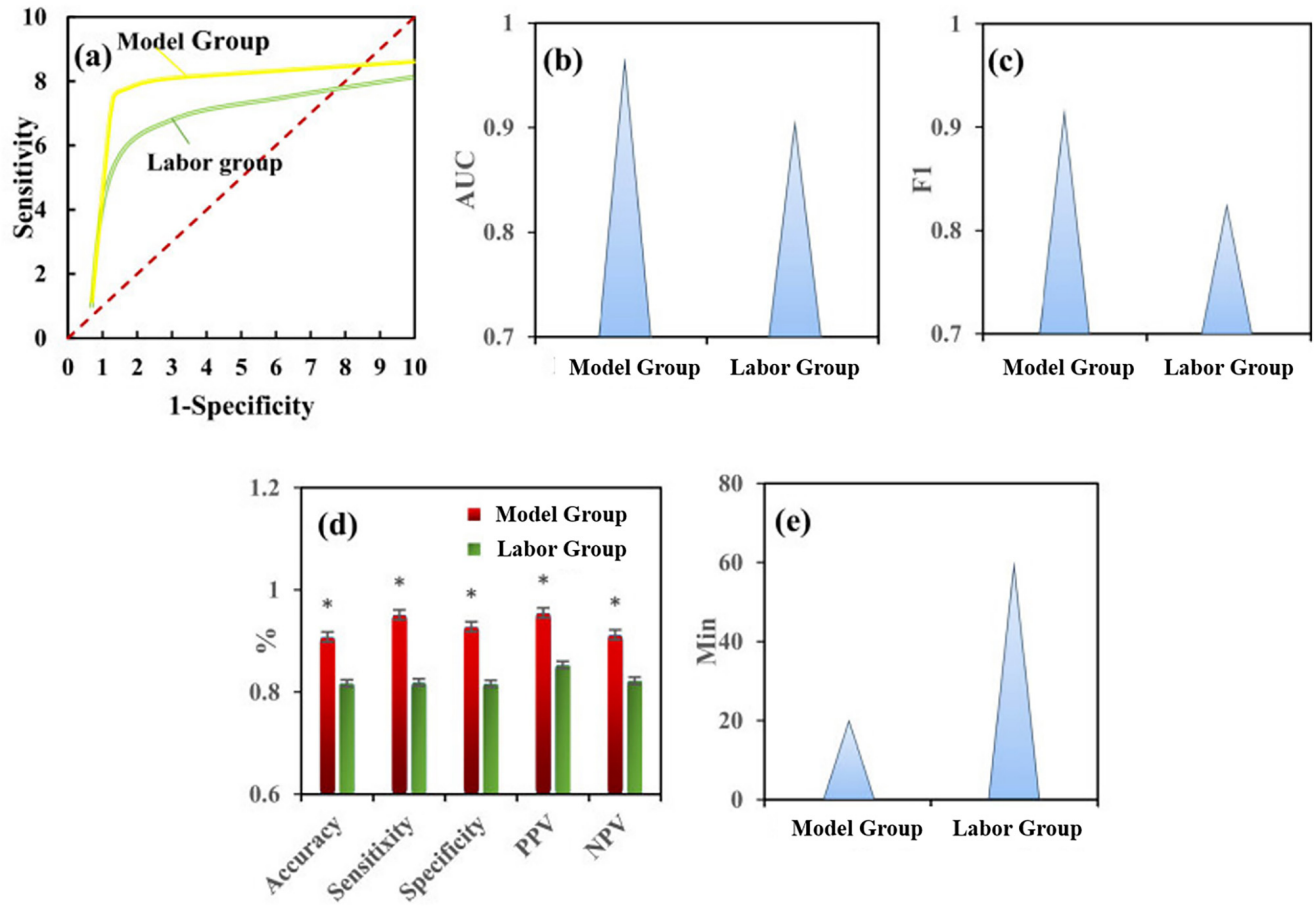
Corrected version of Figure 6 in the article.

